# Assessment of Impacts Produced by Anthropogenic Sources in a Little City near an Important Industrial Area (Modugno, Southern Italy)

**DOI:** 10.1155/2013/150397

**Published:** 2013-02-10

**Authors:** Martino Amodio, Gianluigi de Gennaro, Annalisa Marzocca, Livia Trizio, Maria Tutino

**Affiliations:** ^1^LEnviroS srl, University of Bari, Via Orabona 4, 70126 Bari, Italy; ^2^Chemistry Department, University of Bari, Via Orabona 4, 70126 Bari, Italy

## Abstract

An annual monitoring campaign of VOCs, consisting of twelve sampling periods, was carried out from June 2008 to June 2009 in Modugno, a city located in the Apulia region (Southern Italy), in order to assess the urban air quality, identify the main emission sources, and quantify the cancer and no-cancer risk attributable to inhalation exposures. Monitoring, carried out by using the Radiello diffusive samplers, was conducted in eleven sampling sites throughout the city taking into account the traffic density and the architecture of the city. From the study of the data, it was found that, among all considered VOCs, benzene, toluene, ethylbenzene, and xylenes (BTEX) are the pollutants at higher concentration. The analysis of VOC concentrations, the study of the topography of the city, and the use of different diagnostic ratios between the BTEX species showed that the vehicular traffic emissions were the predominant source of VOCs in the urban area of Modugno. Despite that the annual concentration of benzene is lower than the regulatory limit, the estimation of cancer risk showed that the global lifetime cancer risk attributed to the investigated VOC exposure was not negligible and therefore should be taken into account in future regulatory approaches.

## 1. Introduction

Because of their considerable impact on the environment and human health, volatile organic compounds (VOCs) are considered essential parameters for assessing the air quality in indoor and outdoor environments. A large number of VOCs are usually emitted into the atmosphere of industrialized countries as discussed in different studies [1–4]. VOCs play a significant role in the formation of oxidants like ozone and peroxyacetyl nitrate (PAN) in the troposphere and secondary aerosols [5]. Although motor vehicles are the major source of VOCs in urban areas, these pollutants are also emitted from chemical plants, petroleum refineries, dry cleaning establishments, filling stations, painting operations, and even many household products [6]. Benzene, toluene, ethylbenzene, and xylenes (BTEX) are generally compounds associated with traffic emissions; toluene is also released with the use of solvents (painting, printing, dry cleaning, etc.) as discussed in many works [7–9]. On a global scale, the biogenic VOC emissions, mainly isoprene, *α*-pinene and limonene, dominate over the anthropogenic sources [10, 11]. In and around urban areas, anthropogenic emissions of VOCs are usually more significant [12–15]. The chemical diversity of the VOC group is reflected in the diversity of the health effects that individual VOCs can cause, ranging from relatively inert VOCs with no known health effects to the highly toxic effects of reactive VOCs [4, 16, 17]. In general, chronic health effects of VOCs can be classified as either non-carcinogenic or carcinogenic. The main noncarcinogenic chronic effects are irritative, sensory effects, damage to the liver, kidneys, and central nervous system, asthma, and other respiratory effects. The main carcinogenic effects are lung, blood (leukaemia and non-Hodgkin's lymphoma), liver, kidney, and biliary tract cancer. Most researches have focused on the urban levels of VOCs, especially aromatic and chlorinated organic compounds, due to the known and suspected carcinogenic nature of these species [4, 16, 17]. In fact, several VOCs found in urban air are accepted carcinogens (1,3-butadiene, benzene, formaldehyde, and acetaldehyde). The Organisation for Economic Co-operation and Development (OECD) classified these compounds according to their cancer risk. Benzene and 1,3-butadiene account for 68% of the cancer risk from all vehicle related pollutants, whereas in comparison, particulate matter accounts for only 28% [18]. Benzene has been shown to cause cancer in both animals and humans; therefore, it is currently classified by the Environmental Protection Agency (EPA), the American Conference of Governmental Industrial Hygienist (ACGIH), and the International Agency for Research on Cancer (IARC) as a human carcinogen [19–21]. In European countries, only the ambient concentration of benzene, limited to an annual average of 5 *μ*g/m^3^, is regulated [22]. Other alkylbenzenes cannot be classified as a carcinogen compounds, since there is inadequate evidence for them carcinogenicity in humans even if many studies have shown the several effects linked to the exposure to these pollutants [23–27]. 

In the present study, an annual monitoring campaign of VOCs was performed in eleven sampling sites of Modugno, a city located in Apulia region (southern Italy), in order to assess the urban air quality, identify the main emission sources, and quantify the cancer and no-cancer risk attributable to inhalation exposures. Moreover, a comparison of the monitored VOC concentrations with the levels detected in other Apulia cities and other urban areas was conducted. 

## 2. Materials and Methods

### 2.1. Sampling Sites

**Figure 1:**
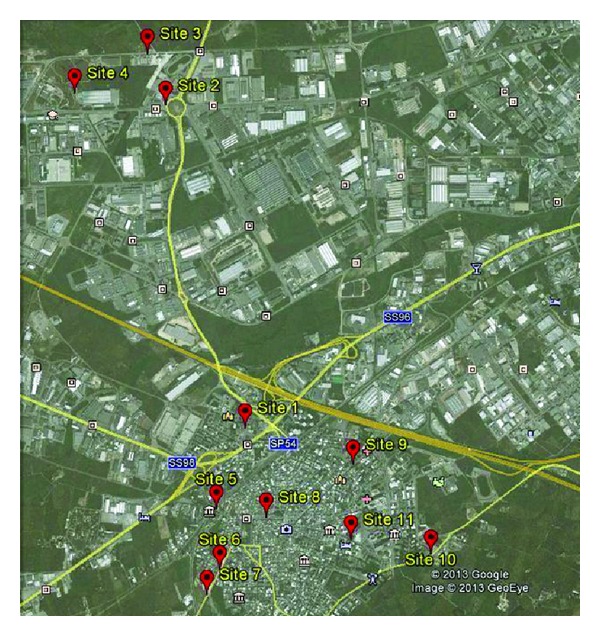
Map of Modugno: sampling sites.

An annual monitoring campaign of VOCs was carried out from June 2008 to June 2009 in Modugno, a town located in Apulia region (Southern Italy). The samples were collected in eleven sampling sites throughout the city for a week at month (Figure 1) to make a detailed characterization of the composition of Modugno urban air. 

Since the 1970s, this town was mainly dedicated to agriculture but after the construction of the industrial zone, it has become one of the most important industrial sites of the region. The industrial area of Modugno is characterized by the presence of national and international enterprises dealing with mechanical, transport, logistic, chemical, and pharmaceutical activities; close to the city, there is also a natural gas energy plant production. The selection of the sites was planned taking into account the traffic density and the architecture of the city: the sites were positioned in order to be as representative as possible of the mean concentration levels of pollutants for each area. In particular, the sampling sites were divided among hot-spot points (4 locations mainly associated with main roads where high traffic density was identified) and 7 points of background indicative of the levels of exposure of not near to an intense circulation of traffic. 

### 2.2. Sampling Method

VOCs were sampled with Radiello diffusive samplers (Fondazione Salvatore Maugeri, Padova, Italy) suitable for thermal desorption. The sampling system is made up of a cylindrical adsorbing cartridge housed coaxially inside a cylindrical diffusive body of polycarbonate and microporous polyethylene. The cartridges are composed of a cylindrical stainless steel net (100 mesh) with the external diameter of 4.8 mm, containing 350 mg of 35–50 mesh of Carbograph 4. Before the sampling, the cartridges were conditioned and analysed to verify the blank levels [28]. Each sampler was exposed and after sampling it was sealed in a sealed glass tube and brought to the laboratory for the analysis. Contemporarily the main meteorological parameters (ambient temperature, wind, atmospheric pressure, and natural radioactivity) were monitored. 

### 2.3. Analytical Method

The analyses were carried out by using a thermal desorber (Markes International Ltd., Unity) equipped with an autosampler [Markes mod. ULTRA TD] provided with 100 positions and coupled with a gas chromatograph (Agilent GC-6890 PLUS) and a mass selective detector (Agilent MS-5973 N). The thermal desorber provides a two-stage mechanism: in the former the analytes are desorbed from the sample tube and refocused into a cold trap; in the latter they are desorbed from the trap and carried into the GC column [28]. The parameters of thermal desorption and GC-MS analysis are listed in Table 1. The standard solutions were prepared by successive dilution in methanol of a VOC standard mixture at 2000 *µ*g/mL (Ultra scientific Cus-5997). To quantify the samples, the calibration curves were prepared injecting 1 *µ*L of the standard solutions into a tube; the spiked adsorbent tubes were then thermally desorbed in the same conditions of time, gas flow, and split ratio of the samples. The sampling rates, *Q* values supplied by manufacturer, were useful to calculate the real concentration of compound in the atmosphere (*C*) by GC quantification of analytes mass,  *m*. *Q*  is function of the diffusive coefficient *D*, which is a thermodynamic property of each chemical substance. The sampling rate has the dimensions of a gaseous flow: if *m* is expressed in *µ*g, the sampling period in minutes and *C* in *µ*g*·*L^−1^, *Q* is expressed in L*·*min⁡^−1^.

## 3. Results and Discussions

Annual mean concentrations (*µ*g/m^3^) of each monitored compound were calculated in all investigated sites and are listed in Table 2. The sum of the detected compounds (VOCSum) and sum of benzene, toluene ethylbenzene, and xylenes (BTEX) in each sampling site are also reported. From Table 1, it can be observed that, among all considered VOCs, BTEX are the pollutants at higher concentration in all sites. Mean BTEX/VOCSum ratio percentage over the city was equal to 63 and the highest ratios were obtained for sites 1, 5, and 8. 

**Figure 2:**
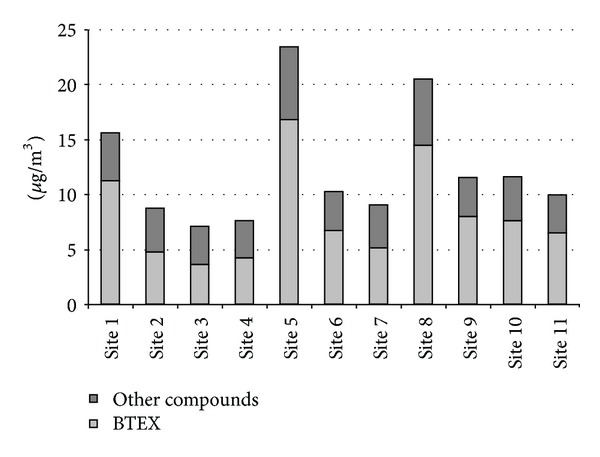
Trend of VOCSum, differentiated among BTEX and other compounds, in all monitored sites.

**Figure 3:**
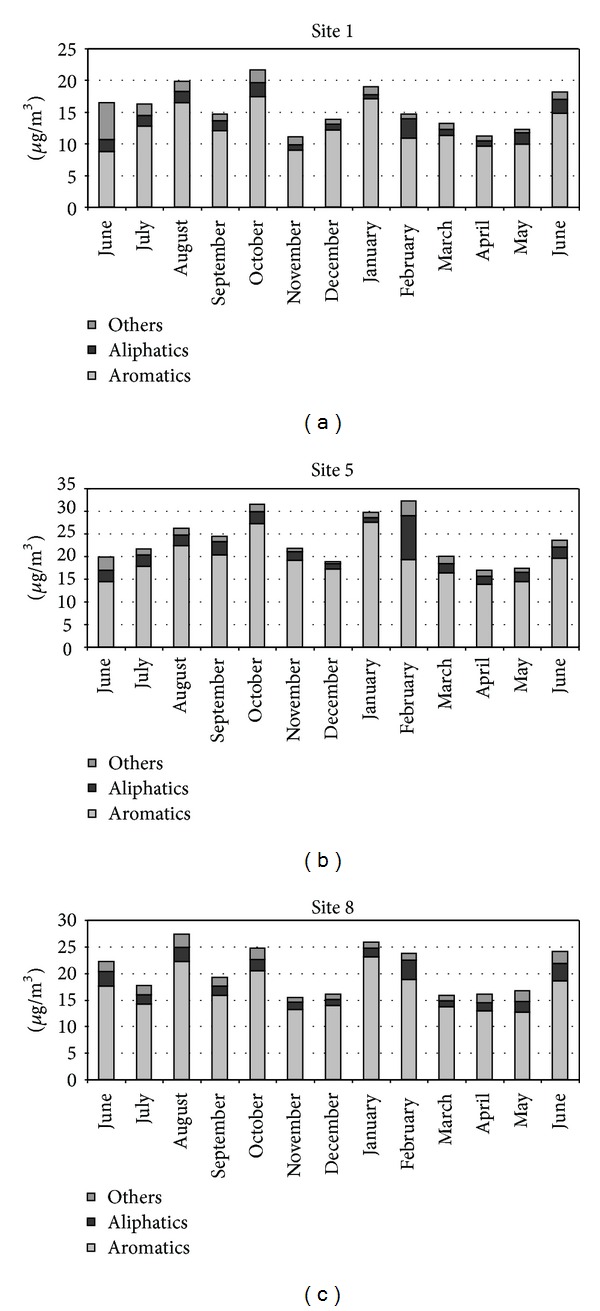
Trend of VOCSum, differentiated among aromatics aliphatics and other compounds, in the most polluted sites.

This finding is also evident in Figure 2 where the trend of VOCSum, differentiated among BTEX and other compounds, is shown. Nevertheless it was found that the annual benzene concentrations in urban area of Modugno was lower than the limit value for 2009 (6 *µ*g/m^3^) [22]. The study of the topography of the area revealed that site 5 is located in a narrow road with heavy traffic and low speed of travel due to the presence of a level crossing. Moreover, the site is impacted by emissions produced by the main artery of the city characterized by heavy traffic. Site 8 represents a critical area of the city center as it is located in a very busy road with many intersections and traffic lights that slow down the speed of travel. Site 1 was near one of the main access roads to the city with high traffic density. The highest concentrations of VOCs were monitored in August, October, January, and June (see Figure 3). 

Furthermore, high concentrations of decane (1.5–8.0 *µ*g/m^3^) were revealed in most sites in February. Sites 3 and 4, located in a peripheral area of the city and near the industrial area, were characterized by the lowest levels of VOCs and BTEX. It is known that toluene and benzene concentrations are reduced in atmosphere through their reaction with OH radicals with the rate of toluene approximately 5 times larger than that of benzene. Therefore, ambient T/B ratios that are significantly lower than vehicular emission ratios are expected to have travelled and degraded, whereas higher T/B ratios may reflect relatively fresh vehicular emission sources. In the present study, annual values of T/B ratio in Modugno (2.1) were nearly similar to those found in urban areas of many Apulian cities such as in Bari (2.0) and Canosa di Puglia (3.4) (see Table 3) [29, 30].

Similar ratios were observed by other researches in urban sites: in Rome (2.8), Izmir (2.0), Santiago (2.01), Paris (2.9–3.4), and Copenhagen (2.2) [31–35]. The correlation analysis among annual VOC concentrations in all sites was also performed (Table 4). Significant positive correlation coefficients were found among BTEX concentrations (≥0.99) and among aromatic hydrocarbons with the exception of styrene. This finding is indicative that local traffic emissions were large contributors to these compounds. 

**Figure 4:**
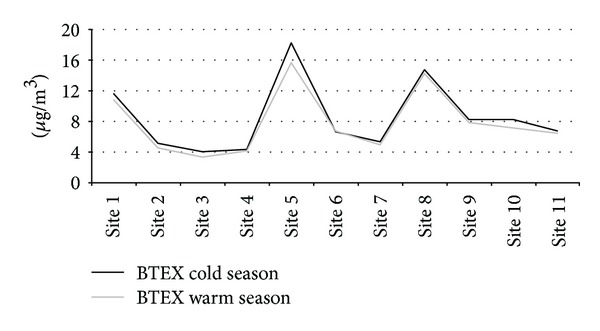
BTEX concentrations during the warm season (April–September) and cold season (October to March).

Several studies identified a group of ratios used as an indicator of the photochemical age of the air mass. In addition to T/B, (m + p)-xylene to ethylbenzene ((m + p)/E), (m + p)-xylene to benzene ((m + p)/B), and o-xylene to benzene (O/B) were also used for such purpose [36, 37]. Higher values of these ratios typically indicate fresh local emissions, whereas lower values are associated with more photochemical degradation and therefore suggest that a sampling site is being influenced by emissions originated some distance away. Values of ratios reported as “high” in a previous study are as follows: 2.45 (T/B), 3.28 ((m + p)/E), 1.61 ((m + p)/B), and 0.85 (O/B) [38]. The average ratios found in the present study were in agreement with those found by Khoder[38]confirming the presence of local emission sources: 2.13 (T/B), 2.988 ((m + p)/E), 1.45 ((m + p)/B), and 0.56 (O/B). Furthermore, B : T : E : X ratios can be useful for comparing sites across a city to identify areas influenced more strongly by one of the emission sources [39]. In most sites, benzene was in lower concentration ratios than would normally be attributable to traffic emissions (3 : 4 : 1 : 4) while lower ratios of xylenes were found in sites 3 and 4 (X/E = 23) located near the industrial area. Analyzing BTEX concentrations during the warm season (April–September) and cold season (October to March), as shown in Figure 4, we can see that the sites are characterized by concentrations approximately constant during the year. BTEX concentrations did not show a seasonal trend. This finding can be explained considering that the high emissions of vehicular traffic, usually close to monitoring sites, contributed predominantly to the levels of VOCs monitored. 

**Figure 5:**
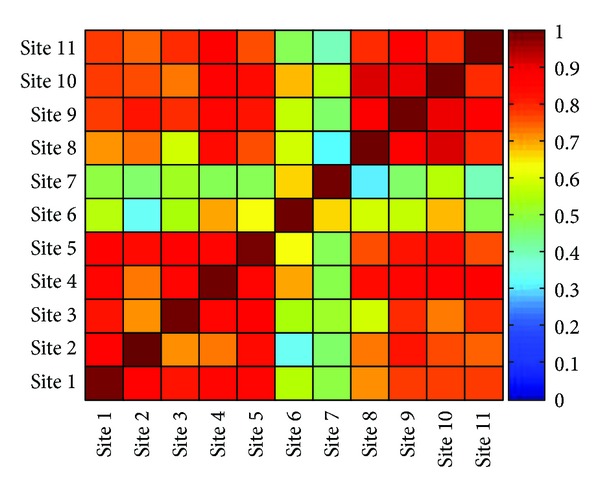
Correlation analysis among BTEX concentrations in all investigated sites.

The correlation analysis among BTEX concentrations in all sites confirmed this hypothesis (Figure 5). A significant positive correlation was found among investigated sites with the exception of sites 6 and 7 characterized by lower correlation coefficients. 

This study focused also on characterizing the risk of exposure to VOCs by means of inhalation in people living in the city. The estimates for the non-cancer risk assessment were based on the comparison of the ambient concentrations with their respective chronic noncancer inhalation level at which no adverse effects are expected for single VOCs. These levels are expressed as Reference Concentrations (RfCs)—used by the USEPA Integrated Risk Information System (IRIS); Minimum Risk Levels (MRLs)—used by the Agency for Toxic Substances and Disease Registry (ATSDR); or Reference Exposure Levels (RELs) used by the California Office of Environmental Health Hazard Assessment (OEHHA) [40–42]. Therefore, the hazard ratio (HR) of each compound was calculated by dividing its concentration (expressed in *μ*g m^−3^) by its corresponding Reference Concentration (RfC, also in *μ*g m^−3^) [24]. The calculated average HRs were less than 1 for all the studied VOCs, which means that their concentrations were commonly below the level of concern (see Table 5). 

Cancer risk assessment was calculated for the carcinogenic VOCs detected in the samples, whose unit risk (UR) values were established by an official agency. These URs were extracted from databases provided by WHO, IRIS, and OEHHA giving priority to the WHO and IRIS URs, in that order (see Table 4) [23, 40, 42]. The lifetime cancer risk (LCR) attributable to inhalation exposures was calculated by multiplying each UR estimated value by the average concentrations (in *μ*g m^−3^) of each compound. The UR of each compound corresponds to excess lifetime cancer risk calculated as a result of the continuous exposure to an agent at a concentration of 1 *μ*g m^−3^ over a lifetime of 70 years [24, 40]. In this study, the individual cancer risk of each VOC was assumed to be additive, and therefore, the global lifetime cancer risk was considered as the sum of the LCRs of the individual compounds. In accordance with previous studies, compounds with an attributable cancer risk of over 10^−4^ can be considered as a “definite risk,” between 10^−5^ and 10^−4^ as a “probable risk” and between 10^−5^ and 10^−6^ as a “possible risk” [24]. Among the monitored compounds, benzene, toluene, tetrachlorethylene, and 1,4-dichlorobenzene are the compounds having the UR estimated value. For each compound, the possible cancer risk for humans was determined (mean LCRs ranged from 7.21 × 10^−7^  to 8.29 × 10^−6^). Moreover, the global lifetime cancer risk was calculated in each site summing the LCRs of the individual compounds (Table 6). 

Similar global lifetime cancer risks were found in the investigated sites: in each one, the estimated risk was higher than the USEPA guideline (10^−6^). This finding showed that the average cancer risk attributed to these VOC exposure was not negligible and therefore should be taken into account to preserve the health of citizens.

## 4. Conclusion

In this study, an annual monitoring campaign of VOCs was performed in eleven sampling sites of Modugno, a city located in Apulia region (southern Italy), in order to assess the urban air quality, identify the main emission sources, and quantify the cancer and no-cancer risk attributable to inhalation exposures. The analysis of VOC concentrations, the study of the topography of the city, and the use of different diagnostic ratios between the BTEX species allowed to identify the vehicular traffic emissions as the main source of pollution in the urban area of Modugno. Despite that the benzene annual concentration is lower than the limit value, the estimating of the cancer risk showed that the global lifetime cancer risk attributed to investigated VOC exposure was not negligible and therefore should be taken into account in future regulatory approaches. In particular, greater attention should be given to VOCs because they can affect air pollution and the distribution of cancer and non-cancer health risks.

## Figures and Tables

**Table 1:** Annual mean concentrations of the investigated pollutants in each sampling sites expressed as *μ*g/m^3^.

Step	Parameter	Value
Cartridge conditioning	Conditioning time	15 min
	Conditioning temperature	310°C
	Gas flow	1 mL/min
	Split flow	50 mL/min

	Purge time	1 min—trap in line
	Desorption time	10 min
Adsorbent tube desorption	Desorption temperature	300°C
	Temperature of cold trap	−10°C
	Desorption flow	20 mL/min

Focusing trap desorption	Desorption time	3 min
	Temperature of cold trap desorption	300°C
	Split flow	44 mL/min
	Temperature transfer line	150°C

GC analysis	Gas carrier	He
	Gas flow	1.7 mL/min
	Analytical column	Polyethylene glycol 30 m × 0.25 mm ID, 0.25 *μ*m
	SUPELCOWAX (Supelco)	Stationary phase thickness
		40°C per 3 min, 8°C/min fino a 80°C
	Oven temperature	80°C per 1 min, 20°C/min fino a 270°C
		270°C per 3 min.

**Table 2:** Annual mean concentrations of the investigated pollutants in each sampling site expressed as *μ*g/m^3^.

	sito 1		sito 2		sito 3		sito 4		sito 5		sito 6		sito 7		sito 8		sito 9		sito 10		sito 11	
	*μ*g/m^3^	SD	*μ*g/m^3^	SD	*μ*g/m^3^	SD	*μ*g/m^3^	SD	*μ*g/m^3^	SD	*μ*g/m^3^	SD	*μ*g/m^3^	SD	*μ*g/m^3^	SD	*μ*g/m^3^	SD	*μ*g/m^3^	SD	*μ*g/m^3^	SD
Benzene	1.83	0.71	1.00	0.48	0.88	0.42	0.89	0.48	2.57	1.00	1.21	0.55	0.89	0.43	2.14	0.81	1.28	0.52	1.38	0.64	1.11	0.47
Heptane	1.03	0.40	0.61	0.32	0.49	0.22	0.90	1.51	1.53	0.69	1.01	1.27	0.66	0.31	1.33	0.51	0.89	0.40	0.93	0.73	0.93	0.71
Toluene	4.22	1.01	1.79	0.72	1.34	0.44	1.63	0.55	6.27	1.59	2.50	0.75	1.89	0.71	5.32	1.11	3.19	1.00	2.78	0.68	2.64	1.37
Tetrachloroethylene	0.38	0.10	0.30	0.08	0.27	0.07	0.29	0.10	0.35	0.11	0.29	0.09	0.29	0.12	0.88	0.43	0.59	0.14	0.45	0.15	0.38	0.12
Butyl acetate	0.33	0.18	0.25	0.11	0.27	0.14	0.28	0.15	0.34	0.13	0.20	0.09	0.23	0.23	0.28	0.09	0.26	0.11	0.19	0.07	0.22	0.09
Ethylbenzene	0.95	0.26	0.42	0.18	0.33	0.12	0.38	0.15	1.41	0.36	0.57	0.14	0.43	0.19	1.24	0.25	0.68	0.22	0.65	0.15	0.52	0.18
m-, p-Xylenes	3.00	0.71	1.14	0.53	0.79	0.26	0.94	0.31	4.70	1.09	1.71	0.46	1.37	1.17	4.12	0.92	2.06	0.72	2.02	0.52	1.61	0.65
o-Xylene	1.18	0.30	0.43	0.18	0.30	0.10	0.34	0.10	1.87	0.46	0.68	0.21	0.52	0.48	1.62	0.34	0.78	0.25	0.79	0.21	0.62	0.23
Styrene	0.20	0.07	0.19	0.12	0.16	0.11	0.31	0.31	0.23	0.04	0.17	0.05	0.30	0.35	0.22	0.07	0.19	0.10	0.17	0.06	0.18	0.07
a-Pinene	0.69	1.35	0.33	0.13	0.42	0.39	0.55	0.46	0.43	0.56	0.36	0.23	0.49	0.82	0.31	0.22	0.29	0.20	0.51	0.36	0.40	0.55
1,3,5-Trimethylbenzene	0.28	0.12	0.14	0.07	0.10	0.05	0.10	0.05	0.54	0.26	0.21	0.15	0.21	0.33	0.48	0.24	0.21	0.12	0.23	0.13	0.20	0.11
2-Ethyl-1-hexanol	0.11	0.19	0.18	0.22	0.26	0.36	0.19	0.24	0.37	0.65	0.18	0.32	0.30	0.49	0.16	0.29	0.11	0.20	0.14	0.32	0.07	0.13
Decane	0.34	0.45	1.31	2.42	1.00	1.95	0.24	0.13	0.94	2.12	0.31	0.25	0.67	1.42	0.50	0.61	0.24	0.22	0.36	0.41	0.26	0.12
1,4-Dichlorobenzene	0.07	0.03	0.06	0.04	0.06	0.03	0.06	0.03	0.07	0.03	0.06	0.03	0.06	0.03	0.07	0.03	0.07	0.03	0.07	0.03	0.06	0.03
1,2,4-Trimethylbenzene	0.47	0.44	0.21	0.20	0.15	0.11	0.16	0.13	1.08	0.74	0.33	0.20	0.33	0.71	0.98	0.75	0.32	0.24	0.41	0.28	0.31	0.26
Limonene	0.19	0.27	0.19	0.13	0.14	0.12	0.23	0.18	0.24	0.33	0.17	0.19	0.21	0.31	0.21	0.23	0.14	0.14	0.18	0.19	0.14	0.11
1,2,3-Trimethylbenzene	0.32	0.36	0.16	0.17	0.15	0.18	0.12	0.12	0.51	0.40	0.27	0.32	0.20	0.24	0.59	0.62	0.25	0.35	0.31	0.35	0.24	0.32

VOCSum	15.59	6.96	8.70	6.10	7.09	5.07	7.63	4.99	23.45	10.56	10.25	5.30	9.05	8.34	20.47	7.51	11.55	4.96	11.59	5.28	9.90	5.51
BTEX	11.18	2.99	4.78	2.09	3.63	1.34	4.18	1.59	16.81	4.50	6.67	2.11	5.11	2.97	14.43	3.43	8.00	2.71	7.62	2.20	6.51	2.90
Percentage (BTEX/VOCSum)	72		55		51		55		72		65		56		71		69		66		66	

**Table 3:** Diagnostic ratios between the BTEX compounds.

Sites	T/B	(m + p)/B	O/B	(m + p)/E	B : T : E : X
Site 1	2.30	1.64	0.64	3.16	2 : 4 : 1 : 4
Site 2	1.79	1.14	0.43	2.69	2 : 4 : 1 : 4
Site 3	1.52	0.89	0.34	2.40	2 : 4 : 1 : 3
Site 4	1.82	1.05	0.38	2.48	2 : 4 : 1 : 3
Site 5	2.44	1.83	0.73	3.32	2 : 4 : 1 : 4
Site 6	2.06	1.41	0.56	3.01	2 : 4 : 1 : 4
Site 7	2.12	1.54	0.58	3.16	2 : 4 : 1 : 4
Site 8	2.49	1.93	0.76	3.32	2 : 4 : 1 : 4
Site 9	2.48	1.61	0.61	3.03	2 : 4 : 1 : 4
Site 10	2.01	1.46	0.57	3.11	2 : 4 : 1 : 4
Site 11	2.38	1.45	0.56	3.09	2 : 4 : 1 : 4

Mean	2.13	1.45	0.56	2.98	
RSD%	15	22	24	11	
Median	2.12	1.46	0.57	3.09	
Min	1.52	0.89	0.34	2.40	
Max	2.49	1.93	0.76	3.32	

**Table 4:** Correlation coefficients among VOC concentrations in all investigated sites: coefficients with values greater than 0.5 are in bold.

	Benzene	Heptane	Toluene	Tetrachloroethylene	Butyl acetate	Ethylbenzene	m-, p-Xylenes	Styrene	a-Pinene	1,3,5-Trimethilbenzene	2-Ethyl-1-hexanol	Decane	1,4-Dichlorobenzene	1,2,4-Trimethylbenzene	Limonene	1,2,3-Trimethylbenzene
Benzene	1.00	**0.91**	**0.99**	**0.51**	**0.62**	**0.99**	**0.99**	−0.04	0.01	0.95	0.23	0.03	0.88	0.95	0.49	0.93
Heptane		1.00	**0.92**	0.49	0.46	**0.91**	**0.91**	0.12	−0.02	0.90	0.13	−0.25	0.79	0.89	0.54	0.88
Toluene			1.00	**0.56**	**0.60**	**1.00**	**1.00**	0.00	−0.04	0.96	0.18	−0.05	0.87	0.95	0.46	0.94
Tetrachloroethylene				1.00	0.13	**0.57**	**0.58**	−0.07	−0.39	0.56	−0.33	−0.27	0.67	0.58	0.06	0.71
Butyl acetate					1.00	**0.61**	**0.58**	0.29	0.32	0.49	0.33	0.20	0.51	0.51	0.49	0.40
Ethylbenzene						1.00	**1.00**	0.02	−0.03	0.97	0.22	−0.01	0.89	0.97	0.50	0.95
m-, p-Xylenes							1.00	0.02	−0.04	0.98	0.22	−0.02	0.89	0.97	0.49	0.96
Styrene								1.00	0.32	0.07	0.40	−0.11	0.04	0.09	0.70	−0.05
a-Pinene									1.00	−0.12	0.02	−0.25	0.11	−0.13	0.31	−0.17
1,3,5-Trimethilbenzene										1.00	0.32	0.05	0.85	1.00	0.51	0.97
2-Ethyl-1-hexanol											1.00	0.60	0.16	0.34	0.51	0.15
Decane												1.00	−0.19	0.08	0.14	−0.05
1,4-Dichlorobenzene													1.00	0.86	0.43	0.90
1,2,4-Trimethylbenzene														1.00	0.54	0.97
Limonene															1.00	0.42
1,2,3-Trimethylbenzene																1.00

**Table 5:** Noncancer reference concentrations. cancer unit risks of the VOCs found during the monitoring campaign and their carcinogenic classifications in the IARC. The mean hazard ratio (HR) and the lifetime cancer risk (LCR) of each compound was calculated.

VOCs	Noncancer		Cancer				
	Reference concentration (*μ*g/m^3^)	Source	Group IARC	Unit risk (*μ*g/m^3^)^−1^	Source	HRs	LCRs
Benzene	9.6	ATSDR	1	6.0*E* − 06	WHO	1.44*E* − 01	8.29*E* − 06
Heptane	—						
Toluene	5000	IRIS	3			6.10*E* − 04	
Tetrachloroethylene	271	ATSDR	2A	5.9*E* − 06	OEHHA	1.50*E* − 03	2.39*E* − 06
Butyl acetate	—						
Ethylbenzene	1300	ATSDR	2B	2.5*E* − 06	OEHHA	5.31*E* − 04	1.72*E* − 06
m-, p-Xylenes	217	ATSDR	3			9.83*E* − 03	
o-Xylene	217	ATSDR	3			3.82*E* − 03	
Styrene	852	ATSDR	2B			2.48*E* − 04	
a-Pinene	—						
1,3,5-Trimethilbenzene	6	PPRTV				4.10*E* − 02	
2-Ethyl-1-hexanol	—						
Decane	—						
1,4-Dichlorobenzene	60	ATSDR	2B	1.1*E* − 05	OEHHA	1.09*E* − 03	7.21*E* − 07
1,2,4-Trimethylbenzene	7	PPRTV				6.15*E* − 02	
Limonene	—						
1,2,3-Trimethylbenzene	5	PPRTV				5.68*E* − 02	

IRIS: Integrated Risk Information System; ATSDR: Agency for Toxic Substances and Disease Registry; PPRTV: Provisional Peer Reviewed Toxicity Values of IRIS; OEHHA: Office of Environmental Health Hazard Assessment; WHO: World Health Organization.

**Table 6:** The lifetime cancer risk (LCR) of each compound and the global lifetime cancer risk in each investigated site.

Sites	Benzene LCR	Tetrachloroethylene LCR	Ethylbenzene LCR	1,4-Dichlorobenzene LCR	Global lifetime cancer risk
Site 1	1.10*E* − 05	2.26*E* − 06	2.37*E* − 06	7.66*E* − 07	1.64*E* − 05
Site 2	5.99*E* − 06	1.77*E* − 06	1.06*E* − 06	6.38*E* − 07	9.45*E* − 06
Site 3	5.29*E* − 06	1.59*E* − 06	8.17*E* − 07	6.96*E* − 07	8.40*E* − 06
Site 4	5.36*E* − 06	1.68*E* − 06	9.50*E* − 07	6.88*E* − 07	8.69*E* − 06
Site 5	1.54*E* − 05	2.09*E* − 06	3.53*E* − 06	7.94*E* − 07	2.18*E* − 05
Site 6	7.29*E* − 06	1.69*E* − 06	1.42*E* − 06	6.99*E* − 07	1.11*E* − 05
Site 7	5.35*E* − 06	1.71*E* − 06	1.09*E* − 06	6.90*E* − 07	8.84*E* − 06
Site 8	1.28*E* − 05	5.20*E* − 06	3.10*E* − 06	8.22*E* − 07	2.20*E* − 05
Site 9	7.71*E* − 06	3.45*E* − 06	1.71*E* − 06	7.21*E* − 07	1.36*E* − 05
Site 10	8.29*E* − 06	2.65*E* − 06	1.62*E* − 06	7.51*E* − 07	1.33*E* − 05
Site 11	6.66*E* − 06	2.23*E* − 06	1.31*E* − 06	6.68*E* − 07	1.09*E* − 05
